# 

**Published:** 1885-07

**Authors:** 


| | PAGE. |
|---|---|
| **ORIGINAL COMMUNICATIONS:** | |
| Pelvic Abscess in the Female, by William Warren Potter, M. D., Buffalo, N. Y. | 531 |
| The Treatment of the Intestinal Diseases of Infancy, by Charles G. Stockton, M. D., Professor of Materia Medica and Therapeutics, Niagara University | 541 |
| **CLINICAL REPORTS:** | |
| Removal of Bursæ of the Wrist by the Operation of Extirpation, by Charles C. F. Gay, M. D. | 550 |
| An Unusual Case of Fracture of the Skull—Trephining Recovery. By W. S. Tremaine, M. D. | 553 |
| **SOCIETY REPORTS:** | |
| Buffalo Medical and Surgical Association, | 555 |
| Transactions of the Buffalo Obstetrical Society, | 558 |
| Medical Society of the County of Erie | 562 |
| **SELECTIONS:** | |
| Levis's Metallic Splints, for Fracture of lower end of the Radius, by R. J Levis, M D | 564 |
| Concentrated Foods. | 567 |
| Removal of the Uterus with Double Ovariotomy | 568 |
| Excision of the Coecum | 569 |
| The Hydrangea Arborescens | 570 |
| The Relation of Over-Nutrition, after the Acute Fevers of Childhood, to Bone Disease | 570 |
| **EDITORIAL:** | |
| Dangerous Physicians | 570 |
| Cocaine as an Antidote for Morphia | 572 |
| **REVIEWS:** | |
| Handbook of Diseases of the Skin. Edited by H. Von Ziemssen, M. D. | 573 |
| The Science and Art of Surgery. A Treatise on Surgical Injuries, Diseases and Operations. By John Erichsen, F.R.S., LL.D., F.R.C.S. | 574 |
| Wormley's Micro Chemistry of Poisons By Theodore G. Wormley, M. D., Ph.D., LL D. | 575 |
| A Manual of Organic Materia Medica. By John M. Maisch. | 575 |
| Insanity and Allied Neuroses Practical and Clinical. By Geo H. Savage, M.D | 576 |
| Modern Medical Therapeutics By George H. Napheys, A M., M. D. | 576 |
| The London Medical Student and other Comicalities. Selected and compiled by Hugo Erickson, M. D | 577 |
| Elements of Practical Medicine. By Alfred H. Carter, M. D. | 577 |
| Hay Fever. By Charles E. Sajous, M. D. | 577 |
| Berlin as a Medical Center. By H. R. Bigelow, M. D. | 578 |
| Sanitary Suggestions on How to Disinfect our Homes. By B W. Palmer, M. D. | 578 |
| A Pharmacopœia for the Treatment of Diseases of the Larynx, Pharynx and Nasal Passages. By George M. Lefferts, A. M., M. D. | 578 |

